# Major depressive disorder as a nonlinear dynamic system: bimodality in the frequency distribution of depressive symptoms over time

**DOI:** 10.1186/s12888-015-0596-5

**Published:** 2015-09-18

**Authors:** Bettina Hosenfeld, Elisabeth H. Bos, Klaas J. Wardenaar, Henk Jan Conradi, Han L. J. van der Maas, Ingmar Visser, Peter de Jonge

**Affiliations:** University of Groningen, University Medical Center Groningen, Interdisciplinary Center Psychopathology and Emotion regulation (ICPE), PO Box 30.001, 9700 RB Groningen, The Netherlands; University of Amsterdam, Department of Psychology, Clinical Psychology, Amsterdam, The Netherlands; University of Amsterdam, Department of Psychology, Psychological Methods, Amsterdam, The Netherlands; University of Amsterdam, Department of Psychology, Developmental Psychology, Amsterdam, The Netherlands

## Abstract

**Background:**

A defining characteristic of Major Depressive Disorder (MDD) is its episodic course, which might indicate that MDD is a nonlinear dynamic phenomenon with two discrete states. We investigated this hypothesis using the symptom time series of individual patients.

**Methods:**

In 178 primary care patients with MDD, the presence of the nine DSM-IV symptoms of depression was recorded weekly for two years. For each patient, the time-series plots as well as the frequency distributions of the symptoms over 104 weeks were inspected. Furthermore, two indicators of bimodality were obtained: the bimodality coefficient (BC) and the fit of a 1- and a 2-state Hidden Markov Model (HMM).

**Results:**

In 66 % of the sample, high bimodality coefficients (BC > .55) were found. These corresponded to relatively sudden jumps in the symptom curves and to highly skewed or bimodal frequency distributions. The results of the HMM analyses classified 90 % of the symptom distributions as bimodal.

**Conclusions:**

A two-state pattern can be used to describe the course of depression symptoms in many patients. The BC seems useful in differentiating between subgroups of MDD patients based on their life course data.

## Background

Major Depressive Disorder (MDD) has been described to occur in episodes [1]. When MDD is presented schematically, its time course is often displayed as a rectangular on-off-curve [2, 3]. Furthermore, patients diagnosed with depression report that they experience their normal state and episodes of depression as discrete mood states [4]. Therefore, depression might be modelled as a nonlinear dynamic system that is attracted to two different states of behaviour and that at times moves from one state to the other. This hypothesis has already been proposed by several authors [5–9], but has not yet been systematically investigated empirically. In one study [9], critical slowing down as an indicator of nearby tipping points predicted mood shifts in depressed patients. This finding provides some indirect evidence for the presence of alternating stable states in depression.

A number of key concepts in the dynamic approach to depression require introduction. Systems are sets of elements interacting with each other. Systems tend to seek an equilibrium, a state of behaviour that minimises energy, in which they can remain as long as there is no compulsion to change. Systems that have equilibria at several levels occasionally switch between them [10]. Depression in individuals might be interpreted as a state of mood, cognitions, and behaviour in which costs are minimised, when, for example, conflicting social demands exceed the perceived resources of an individual [11, 12] or when participating in a joint enterprise does not pay off but cannot be avoided either [13]. In a dynamic system, dependencies exist over time: the behaviour of the system at a certain point in time does not only depend on external parameters, but also on its history. In general, negative mood in a certain period is related to mood in the prior period, while such inertia seems to be stronger in persons diagnosed with depression than in healthy controls [14, 15]. This can be explained by continuously accumulating effects, which have been found to account for about one third of the variance of the depressive symptoms in young adults [16]. Moreover, internal or external influences, referred to as control parameters, can change the behaviour of a system. Chronic stress, for instance, is a risk factor for depressed mood [17]. In a nonlinear system, the relationship between the control parameters and the behaviour of the system is more complex. Small continuous changes of a control parameter can sometimes lead to large and rapid changes in the behaviour of the system. The dynamic state of the system explains this phenomenon; a system in equilibrium will hardly respond to a small perturbation, while a system near a transition will respond vehemently. In most people, a small disagreement or a bad joke will not change their mood lastingly. After a period of cumulative hassles, however, the same trigger might have a stronger impact on their mood.

Some systems are more prone to rapid change than others [10]. The structure of these highly vulnerable systems is characterised by high homogeneity and high connectivity. Homogeneity means that a system consists of similar elements; connectivity means that connections between the elements allow mutual influence and strong positive feed-back loops. Highly homogeneous and connected systems can easily resist small perturbations. When one of the elements fails, its function will be taken over by one of the similar neighbours. Such a system, however, is less able to adapt to a continuously changing environment. Change will happen in leaps. Given its episodic temporal structure, MDD is likely to be such a highly homogeneous and connected system. The recent development of defining the symptoms of depression as highly connected nodes of a network [7, 18] is in line with this, as high connectivity can explain the observed patterns of distinguishable episodes. Because activation as well as de-activation can spread quickly through the network of symptoms, the network is observed in either one of two discernible states: either most of the elements are activated and nearly all symptoms are present, or most of the elements are deactivated and nearly all symptoms are absent. Consequently, the transitions from a normal mental state to a state of depression and vice versa can take place rapidly.

If a system can adopt two separate states of behaviour, two discernible modes should be observable in the frequency distribution that displays the temporal behaviour of the system. If depression and normal mental state constitute two qualitatively different states, then one should find bimodality in the frequency distribution of the symptoms reported over time (cf. [19]).

The aim of this preliminary proof-of-concept study was to investigate whether depression can be viewed as a nonlinear dynamic system moving between two equilibrium states. We explored a longitudinal dataset for evidence of two discrete states, symptom-free versus depressed, in patients with MDD. We hypothesised sudden jumps in the symptom curves and bimodality in the symptom distributions of the individual patients.

## Methods

### Original study

From 1998 to 2003, 267 patients diagnosed with MDD participated in a randomised controlled trial with follow-ups every three months during three years [20, 21]. The study protocol was approved by the Medical Ethics Committee of the University Medical Center Groningen (UMCG). All participants provided informed consent. A total of 397 patients were referred by 49 GP-practices in the North of the Netherlands. Inclusion criteria were: a history of depression, the absence of life-threatening somatic diseases, and receiving no psychotherapy. Patients were excluded if they were pregnant, had dementia, had bipolar disorder, had a psychotic disorder and/or had a primary diagnosis of alcohol or drug dependence. The referred patients were interviewed using the Composite International Diagnostic Interview (CIDI) to confirm the presence of a major depressive episode and the absence of other psychopathology. Out of the 397 patients, 78 refused to participate and 52 met the exclusion criteria, resulting in a sample of 267 patients (67.3 %). These patients were randomly allocated to four treatment arms: (1) care as usual (CAU) following national general practice guidelines, (2) CAU plus psycho-education program (PEP), (3) CAU + PEP + cognitive behavioural therapy, and (4) CAU + PEP + psychiatric consultation. Previous analyses of the data showed that when the treatment effects of three types of enhanced treatment for MDD were compared with the CAU, none of the enhanced treatment options outperformed CAU after one [20] or three years [22].

When looking at patients’ dynamic development over time (onsets and remissions), at least 499 switches from an episode of depression to a healthy state and vice versa were detected in the profiles of the 267 patients over three years, when using the DSM-IV criteria for a depressive episode [3].

### Participants

Of the 267 participants, 178 patients had complete symptom records for a 2-year follow-up period (104 weeks). These records formed the basis for the analyses reported below. Although some patients were followed for a longer period (up to 3 years), the cut-off of 104 weeks was chosen after inspection of the frequency distributions of responders on each of the time-points. The cut-off of 104 weeks resulted in the inclusion of 178 complete cases (66.7 %), which seemed to be an optimal trade-off between sample-size and follow-up time. In contrast, a cut-off of 156 weeks, for instance, would have resulted in the inclusion of only 121 complete cases (45.3 %).

### Procedure

At study entry, all patients participated in a face-to-face interview. Every three months afterwards, they were interviewed by telephone with a computerized interview based on the depression section of the Composite International Diagnostic Interview (CIDI) [23]. The presence of the nine DSM-IV criteria for depression, depressed mood, diminished interest, eating problems, sleeping problems, psychomotor problems, loss of energy, guilt, cognitive problems, and preoccupation with death/suicidal ideation, was recorded retrospectively for every week in the preceding three months [3].

### Measures

In addition to the CIDI assessments described above, socio-demographic characteristics (e.g. work-status, social status, education) were thoroughly assessed at baseline. During the baseline CIDI, the number of previous depressive episodes, the age of onset, comorbid disorders (dysthymia, social phobia, agoraphobia, panic disorder) and the presence of a lifetime suicide attempt were assessed. Also, the Beck Depression Inventory (BDI) [24] was administered to assess depression severity. At 6-month follow-up, 153 of the 178 patients (86.0 %) filled in the BDI again and 148 patients (83.1 %) filled in the BDI after 1 year. These follow-up measurements were used to investigate recovery.

#### Statistical analysis

For each patient, we displayed the number of depressive symptoms per week in a time-series plot over 104 weeks and plotted the frequency distribution of the symptoms, i.e., collapsed over measurements, as a histogram of 104 scores. We inspected the time series plots broadly, applying the three categories “fluctuating symptoms around a mean”, “continuous decrease and/or increase of the symptoms”, “clear sudden jumps visible”. Similarly, we categorised the histograms as either ”unimodal with a mode at the middle of the scale” or as “unimodal with a mode at one end of the scale” or as “bimodal”. The systematic coding of the plots was conducted by the first author. The time series plots were coded independently from the histograms. No other information was used for coding.

Moreover, for every patient, we computed a bimodality coefficient (BC), which reflects the form of the distribution of the scores. The BC is the sum of the squared skewness of a distribution (s) and 1, divided by the sum of the kurtosis (k) and a correction factor (C) [25, 26]: BC = (s^2 + 1)/(k + C) with C = (3*(n 1)^2)/((n–2)*(n-3)). C varies between 3 and 4, depending on the number of observations n, and corrects for the fact that the kurtosis of a normal distribution equals 3 but is reported as zero [27]. In the current study, n equalled 104 observations per patient, leading to C = 3.089. The BC (range 0–1) for a uniform distribution equals 0.55. Accordingly, a BC < .55 indicates unimodality, whereas a BC > 0.55 indicates bimodality. Highly skewed and two-peaked distributions with short tails have a high BC, while symmetric distributions with only one peak and long tails have a low BC. The BCs of the individual patients can be ordered, compared and related to other variables. Note that the BC, a test for cross-sectional data, does not take into account temporal dependencies in a time series.

In addition, we conducted Hidden Markov Model (HMM) analyses [28, 29], which enable detection of qualitatively different behavioural states in a one- or multidimensional time series of an individual system. HMMs are based on three assumptions. First, the process can be described by one or more discrete states. Second, while there are serial dependencies over time, the state of a system depends only on its state at the previous point in the time series, but not on any earlier states. Third, the states cannot be observed directly. Using the R-package depmixS4 [30], we fitted two Hidden Markov Models, a 1-state model and a 2-state model, to each of the individual time series presuming that the number of symptoms was binomially distributed (*n* = 9, p freely estimated). To compare the model fit of the two models, we applied the Bayesian Information Criterion (BIC). Moreover, for each patient, we defined the distance between the estimated modes as the absolute value of the difference between them.

## Results

### Sample Characteristics

The sample (Table 1) included 122 women (69 %). Age ranged from 17 to 69 years (M = 43.4, SD = 11.5). At study entry, 135 patients (76 %) used antidepressant medication. Forty-six patients (26 %) received care as usual from their general practitioners, 71 patients (40 %) participated in a psycho-educational prevention program (PEP) only, 30 patients (17 %) received PEP and psychiatric consultation, and 31 patients (17 %) received PEP and cognitive behavioral therapy. The mean Beck Depression Inventory score was 19.7 (SD = 9.2), indicating moderate depression severity [24]. The median number of previous episodes was 2 and the mean age of first depression onset was 31.9 years (SD = 13.0). Of the patients, 9.6 % reported a previous suicide attempt in their lifetime. Percentages comorbid psychiatric disorders in the month prior to baseline ranged from 7.3 % for dysthymia to 12.4 % for social phobia. The study sample did not differ notably from the original sample (*n* = 267) with respect to socio-demographic and psychiatric characteristics (Table 1). Of the patients with a 6-month BDI follow-up, 21.1 % showed an increase or no change in BDI score, 66.2 % showed a reduction of at least 25 % and 42.1 % showed a reduction of at least 50 % compared to the baseline BDI-score. Of the patients with a 1-year BDI follow-up, 18.2 % showed an increase or no change, 69.7 % showed a reduction of at least 25 % and 49.2 % showed a reduction of at least 50 % compared to baseline.

**Table 1:** Characteristics of the original sample and the final sample

	Original sample	Final sample
	(*N* = 267)	(*N* = 178)
*Sociodemographics*		
Age, mean (SD)	42.8 (11.3)	43.4 (11.5)
Female, *n* (%)	171 (64 %)	121 (68 %)
Years of education, mean (SD)	12.5 (3.7)	12.5 (3.5)
Married/cohabiting, *n* (%)	172 (64.4 %)	117 (65.7 %)
Paid employment, *n* (%)	161 (60.3 %)	109 (61.2 %)
Number of chronic somatic diseases, median (IQR)	1 (0.5-1.5)	1 (0.5-1.5)
*Treatment arm, n (%)*		
Usual care by general practitioner	72 (27 %)	46 (26 %)
Psycho-education (PEP)	112 (42 %)	71 (40 %)
Psychiatric consultation + PEP	39 (15 %)	30 (17 %)
Cognitive Behavioral Therapy + PEP	44 (16 %)	31 (17 %)
*Psychiatric Characteristics*		
Beck Depression Inventory, mean (SD)	20.1 (9.4)	19.7 (9.2)
Number of previous episodes, median (IQR)	2 (0–4.5)	2 (0–4)
Age at first onset, mean (SD)	31.3 (13.2)	31.9 (13.0)
Antidepressant use, *n* (%)	198 (74 %)	134 (75 %)
Previous suicide attempt, *n* (%)	27 (10.1 %)	17 (9.6 %)
*Past month comorbid psychiatric disorders, n (%)*		
Dysthymia	22 (8.2 %)	13 (7.3 %)
Social phobia	41 (15.4 %)	22 (12.4 %)
Panic Disorder	34 (12.7 %)	19 (10.7 %)
Agoraphobia	22 (8.2 %)	18 (10.1 %)

*SD* Standard deviation, *IQR* interquartile range

The number of symptoms reported by the 178 patients during the 104 weeks ranged from 0 to 9. At the group level, the frequency distribution of the number of symptoms was positively skewed with a mode of zero symptoms (*N* = 18,512, BC = .63). Many of the patients recorded only a few symptoms most of the time, which corresponded to the rapid improvements observed during the first months of the intervention [22].

### Time-series plots

**Fig. 1:**
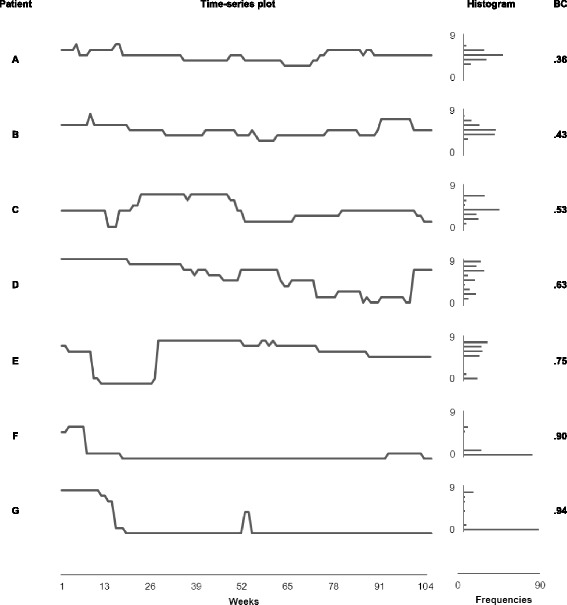
Time-series plots, histograms, and bimodality coefficients for seven MDD patients

For each patient, we displayed the number of depressive symptoms per week as individual time-series plots. Inspecting these plots (Fig. 1), we discovered three main patterns. In 7 % of the plots, the number of symptoms fluctuated within a narrow range. In 38 % of the plots, the number of symptoms continuously decreased as well as increased over time. In the majority of the plots (56 %), one or more clear sudden jumps from many symptoms to nearly no symptoms or vice versa appeared.

### Histograms

Inspecting the histograms that displayed the number of symptoms over 104 weeks for the individual patients, we identified 31 % of the distributions as unimodal either with modes near the extremes of the scale (8 %) or with modes in the middle of the scale (23 %). The majority (69 %) of the histograms either contained two separated modes or was severely skewed. With the exception of the unimodal distributions around the middle of the scale, all distributions reflected the existence of two distinct states, symptom-free versus depressed.

### Bimodality coefficients

To summarise the patterns discovered in the time-series plots and histograms, we computed a BC for each patient. These BCs ranged from .20 to .98 (mean = .65, median = .63, SD = .17), confirming that unimodal, uniform, and bimodal distributions were present in the current sample. The majority (66 %) of distributions was bimodal (BC > .55). The distribution of the BCs did not significantly deviate from normality (Kolmogorov-Smirnov-Z = 0.877, df = 178, *p* = .43). No statistically significant relationships were found between the BC and age (r = −.11, *N* = 178, *p* = .15), gender (t = 0.426, df = 176, *p* = .671), treatment (F = 0.260, df = 3, *p* = .854), or antidepressant use (t = −0.389, df = 176, *p* = .698), respectively.

### Comparison of the bimodality coefficients and graphs

Overall, the BCs for the individual patients effectively summarised the patterns of the symptoms over time. Low BCs corresponded to flat or smooth curves in the time-series plots, while high BCs indicated on-off patterns. Similarly, low BCs were found for the unimodal frequency distributions, while high BCs appeared with skewed of bimodal frequency distributions.

A few time-series plots appeared as nearly flat lines either at the bottom or at the top of the symptom scale; the distribution of the symptoms was unimodal at the extremes of the scale, with BCs between 0.20 and 0.54. The patients who reported these unchanging symptom levels seemed to remain in a single mood state. They might be viewed as potentially highly bimodal individuals who did not go through any phase transition during the two years of observation.

In order to illustrate the relationships between the BCs and the graphs, we blindly selected one case at the 5^th^, the 10^th^, the 25^th^, the 50^th^, the 75^th^, the 90^th^, and the 95^th^ percentile, respectively, of the BC distribution. The individual graphs of these seven cases, ordered by the BC, are displayed in Fig. 1. Obviously, the BC is able to order the time-series plots and the histograms in a comprehensible manner.

### Hidden Markov Models

To each of the individual time series, we fitted two Hidden Markov Models (HMM). Comparing the BIC, we found that a 1-state model best fit the data in only 18 individuals (10 %), whereas the 2-state model outperformed the 1-state model for 160 individuals (90 %). In all patients who were best described by 2-state models, high probabilities to remain in the previous state (p > .87) indicated stable states. The classification by HMM was positively related to the BC (rho = .33, *N* = 178, p < .001). Distributions with a high BC were classified as bimodal, distributions with a low BC as unimodal.

Additionally, the distance between the estimated modes in the 2-state model varied between 0 and 7.98 symptoms and was clearly related to the outperforming model, respectively (rho = .52, *N* = 178, p < .001). Distributions with small distances between the estimated modes were best fit by the 1-state model, whereas distributions with large distances were best fit by the 2-state model. In general, the distance between the estimated modes was also associated with the BC (rho = .44, *N* = 178, p < .001). Small distances between the modes corresponded to low BCs, while large distances corresponded to high BCs. Detailed information about the results of the HMM analyses for the seven prototypical patients can be found in Table 2.

**Table 2:** Goodness of fit for the 1- and 2-state Hidden Markov Models for seven patients

ID	Model	Θ1	Θ2	Θ2 - Θ1	logl	AIC	BIC	nfree	BC
A	1	4.87			−158.89	319.77	322.42	1	.36
	2	4.87	4.87	0.00	−158.89	327.77	340.99	5	
B	1	4.94			−164.52	331.05	333.69	1	.43
	2	4.94	4.94	0.00	−164.52	339.03	352.25	5	
C	1	4.21			−210.47	422.93	425.58	1	.53
	2	3.26	6.81	3.55	−158.53	327.06	340.28	5	
D	1	5.87			−300.57	603.14	605.78	1	.63
	2	3.02	7.73	4.71	−175.20	360.39	373.62	5	
E	1	5.53			−306.42	614.83	617.48	1	.75
	2	0.17	6.65	6.48	−150.89	311.77	325.00	5	
F	1	0.58			−147.10	296.20	298.84	1	.90
	2	0.00	2.20	2.20	−72.26	154.51	167.73	5	
G	1	1.20			−311.46	624.91	627.55	1	.94
	2	0.03	7.18	7.15	−51.94	113.89	127.11	5	

Note. *Θ* mean number of symptoms per mode, *Θ2* Θ1 = distance between the modes, *logl* log likelihood, *AIC* Akaike’s Information Criterion, *BIC* Bayesian Information Criterion, *nfree* number of freely estimated parameters, *BC* Bimodality Coefficient

## Discussion

The present study provides evidence for the idea that depression can be modelled as a nonlinear dynamic system moving between two equilibrium states. We found that, in a majority of 178 patients with MDD, the symptoms of depression were closely inter-related during a period of 104 weeks. At each measurement point, these patients reported either many symptoms or nearly no symptoms at all, while the probability to remain in a given state, depressed or non-depressed, was high. Consequently, decreases as well as increases of their symptoms over time happened rapidly. The minority of the sample reported the depression symptoms to vary more or less randomly around a mean or to change gradually. These patients did not seem to switch as abruptly between mental states. These findings suggest that the dynamics of depression over time differ across patients, with many patients showing more or less abrupt transitions from one state to the other and others showing more continuous variation in depression severity over time. On the one hand, these results support the traditional all-or-nothing distinction between depression and health. On the other hand, the results show that bimodality is a matter of degree and that in many patients continuous temporal variations in depression severity also play an important role. These variations are a potentially important source of between-person heterogeneity and might indicate the involvement of different underlying mechanisms.

Comparing the results of the two different bimodality analyses, BC and HMM, we found that different proportions of the sample (66 % and 90 %, respectively) were classified as bimodal. Although the results of the two analyses were positively associated, the two methods led to different decisions regarding bimodality for several individual distributions. We made two observations: first, the HMM analyses detected bimodality, even if the distances between the estimated modes were rather small. Second, several cases with a high BC were classified as unimodal by the HMM analyses. The symptom distributions of these cases were heavily skewed, but did not contain a visible second mode. Both observations matched the finding that, in general, mixture distributions are more sensitive to small distances between the estimated modes than the BC, but less sensitive to small proportions at large distances [25, 26].

For further exploration of bimodality in MDD patients, we would recommend the BC. First, the BC is related to non-trivial distances between the modes, and thus, possibly to clinically relevant features. Second, the BC is robust against unequal proportions in the modes and obviously corresponds to the time series plots and the histograms. Apparently, it allows arranging patients on a scale that describes the probability that their symptoms emerge and disappear rapidly. Third, the BC does not depend on parameter estimation or on the choice of a basic distribution, but can be computed with a simple formula. With the help of the BC, one might be able to distinguish subgroups of MDD patients and to explore whether they display an increased chance of recurrence of depression, of recovery, or even of experiencing a (hypo)manic state.

Nonlinear dynamic systems move from one state to another under the influence of a small set of control variables; during a phase transition, it is possible to identify those variables that control the system [5]. Therefore, one might first speculate on which variable is involved in a nonlinear dynamic model for depression as a splitting control variable, that is, which force is responsible for the different degrees of bimodality we found. When we explored some possibly associated variables like age, gender, treatment and medication, we did not find any associations with the BC. Another candidate for the splitting control variable might be the structure of the network of depression symptoms itself [7, 10]. If depression is assumed to form a network of highly connected symptoms containing strong positive feed-back loops, depression can be expected to undergo sudden phase transitions. This has already been demonstrated in a NetLogo simulation [31, 32], in which the degree of connectivity in a network of depression symptoms influences the form of its time series. With high connectivity, the symptom network produces time-series plots with clearly rectangular patterns. Thus, in an individual patient, the degree of connectivity of the symptom network would explain the value of the BC. High connectivity would bring about the sudden jumps in the symptom curves and bimodality in the frequency distributions.

Furthermore, one might wonder which control variable is responsible for the switch of the system from one state to the other. This variable presumably represents a perturbing force, such as perceived stress, conflict, or entrapment. Depending on the architecture and the current state of the individual system, the same amount of perturbation can cause either a small or a large effect. We would expect that no single control variable will be able to explain the switch to or from an episode of MDD for all individual patients. Most probably, idiosyncratic control variables will be identified for different persons. In our study, we cannot exclude the possibility that the repeated interviews have triggered the switches from one regime to the other in some individual patients. This might mean that even repeated interviews may function as a perturbing force in MDD.

### Limitations of the study

The current study was intended as a preliminary proof-of-principle study and several limitations should be kept in mind when interpreting the presented results. First, the bimodality pattern we discovered in part might reflect artefacts stemming from the data-collection method. All information about the symptoms was recorded retrospectively over three-month periods. Possibly, the participants of the study reported the onset and remission of the symptoms as more or less simultaneously only because it is easier to recall and to report simple patterns than more differentiated or random ones. Alternatively, the patients might have reported a two-state pattern because it fitted their expectations about depression. Three of the four treatments, however, included the instruction to monitor the onset of depression symptoms. All patients who received enhanced treatment were encouraged to keep diaries about the symptoms they experienced and might have consulted these diaries when responding to the interviewers. Accordingly, one would expect that the subgroup of patients receiving care as usual presented the least differentiated symptom curves. Nevertheless, we found no differences between the symptom patterns of the four treatment groups. Second, we included only complete cases in our analyses. Possibly, the missing values in the original dataset were systematically missing values. Those patients, for example, who did not recover during the intervention, might have been the ones who provided the incomplete records. Third, partly due to the selection procedure, the study sample ended up being relatively small. Fourth, the study was conducted among primary care patients and the results may not be directly generalizable to more severely affected patients with potentially more complex comorbidity and/or treatment resistance. Finally, the current analyses were conducted under the rather strict assumption that the symptom sum score could be seen as a unidimensional representation of underlying depression severity and that between-person differences could be quantified in terms of variations on this dimension.

### Recommendations for further research

Ideally, our results should be replicated in larger samples of patients with MDD, by preference in a multi-centre study. Mood recordings from healthy controls might further contribute to insight in the dynamics of depression. Keeping the participants motivated to record their mood continuously and reliably should be a major aspect of the study design.

Furthermore, it might be useful to register the symptoms of depression prospectively, at the moment at which they are experienced and even more frequently than only once a week so that the risk of study artefacts will be minimized. Small hand-held devices have already been used in order to record symptoms or mood scores several times a day [33]. Similar applications for cell phones might further facilitate registration. A validation study comparing retrospective with prospective recordings of depression symptoms might reveal systematic recall errors.

In order to validate the BC further, one might use it to distinguish time series generated either by a model with or a model without alternating stable states. For this test, the NetLogo model [31] might provide the necessary data.

Importantly, bimodality alone cannot prove that alternative stable states exist in a system, because it might only indicate a sharp response of the system to a control parameter near a threshold value [34]. The demonstration of other indicators of nonlinear dynamics, in particular the demonstration of hysteresis, would strongly support the hypothesis that MDD is a nonlinear dynamic system. The term hysteresis refers to the fact that the shift of the observed behaviour towards or away from an attractor happens at different places on the continuum of the control variable. Hysteresis in MDD would mean that for the recovery of individual patients it is necessary that stress be reduced below the stress-level cut-off that triggered the shift to the depressed state.

## Conclusion

Finally, adopting the model of a nonlinear dynamic system for depression in individual persons might lead to the generation of novel hypotheses. There are several formal indicators that can predict abrupt changes in the behaviour of a nonlinear dynamic system: increased autocorrelations, increased variance and prolonged periods of recovery after a perturbation [10, 9]. With these indicators, one might be able to detect or even predict remission and relapse of depression in the time-series data of individual patients with MDD. In conclusion, the results of the current study might provide a valuable contribution to a new perspective on depression and offer interesting opportunities to investigate the etiology, course and treatment of depression.

## Abbreviations

BC: Bimodality coefficient
BIC: Bayesian Information Criterion
HMM: Hidden Markov Model
PEP: Psycho-educational prevention program
